# Atrial fibrillation in Retinal Artery Occlusions

**Published:** 2025-07-07

**Authors:** Shreyas Melanahalli, Tommy Tran, Clement Ng, Devendra K. Agrawal

**Affiliations:** Department of Translational Research, College of Osteopathic Medicine of the Pacific, Western University of Health Sciences, Pomona, California 91766 USA

**Keywords:** Atrial fibrillation, Branch retinal artery occlusion, Central retinal artery occlusion, Cerebral ischemia, Embolism, Ischemic stroke, Retinal artery occlusion, Stroke, Thromboembolism, Thrombolysis, Transient ischemic attack

## Abstract

Retinal artery occlusions, encompassing central retinal artery occlusion and branch retinal artery occlusion, are acute vascular events that can result in significant and often irreversible vision loss. These ocular emergencies are closely associated with systemic vascular risk factors, including hypertension, diabetes mellitus, advanced age, and cardiovascular comorbidities. Atrial fibrillation (AFib), the most common sustained cardiac arrhythmia, has emerged as a notable contributor to the risk of retinal artery occlusions. The prevalence of AFib is rising, particularly among older adults, paralleling the age-dependent increase in retinal artery occlusion incidence. Recent studies indicate a significant association between AFib and retinal artery occlusions, with AFib conferring an elevated risk for both retinal and cerebral ischemic events. Notably, Patients with retinal artery occlusion exhibit a heightened risk of subsequent AFib, stroke, and thromboembolic complications. The pathophysiological interplay involves embolic phenomena originating from cardiac or carotid sources, with AFib facilitating thrombus formation and embolization to the retinal circulation. Diagnostic advances, including prolonged cardiac monitoring and optical coherence tomography, have improved detection of both AFib and retinal ischemic changes. Despite the lack of a universally accepted treatment for retinal artery occlusions, early interventions such as thrombolysis and hyperbaric oxygen therapy may improve visual outcomes if administered promptly. This manuscript underscores the importance of comprehensive cardiovascular evaluation in patients with retinal artery occlusion, particularly for AFib, to optimize risk stratification, guide management, and reduce the likelihood of recurrent vascular events. Enhanced awareness and interdisciplinary collaboration are critical to improving prognosis and preventing further morbidity in this high-risk population.

## 1. Introduction

Retinal strokes, or retinal artery occlusions (RAO), occur when blood flow to the retina is interrupted, leading to a lack of oxygen and nutrients in the affected area. Central retinal artery occlusion (CRAO) involves a blockage in the central retinal artery, causing widespread retinal ischemia and often severe vision loss. In contrast, Branch retinal artery occlusion (BRAO) affects smaller branches of the retinal artery, resulting in more localized visual deficits. Both conditions share similar pathophysiological mechanisms and are considered ocular emergencies due to their potential to cause permanent vision impairment if not promptly addressed.

Central retinal artery occlusion (CRAO) represents a critical ophthalmic emergency. This condition occurs when blood flow to the retina is suddenly disrupted due to a blockage in the central retinal artery, potentially resulting in acute visual impairment or irreversible loss of sight. CRAO can be classified into four primary subtypes: non-arteritic permanent, arteritic, non-arteritic with cilioretinal artery sparing, and non-arteritic transient. Each subtype presents unique characteristics and requires specific management approaches, underscoring the importance of accurate diagnosis and prompt intervention in CRAO cases [1]. CRAO has an acute, painless and potentially devastating impact on visual acuity [2]. In a recent study, analyzing the relative risk (RR) with confidence intervals (CI) of death after a CRAO was significantly higher than the control patients at multiple different time points measured (2 weeks: RR 2.45, 95% CI 1.46–4.12; 30 days: RR 2.10, 95% CI 1.49–2.97; 1 year: RR 1.78, 95% CI 1.61–1.94; 5 years: RR 1.28, 95% CI 1.23–1.33; 10 years: RR 1.05, 95% CI 1.02–1.07) [3]. With the risk of permanent vision loss and mortality, CRAO are significant due to their role as indicators of broader systemic health issues such as functioning as markers of systemic vascular diseases, association with other health conditions, potential for recurrence and their impact on healthcare systems due to their emergent nature. Most patients with CRAO seek care from eye specialists, with only a small number arriving within the critical 4.5-hour window for potential thrombolysis. This underscores the importance of increasing public awareness about the urgency of symptoms associated with CRAO. Additionally, the cohort demonstrates a high prevalence of systemic comorbidities among these patients, emphasizing the need for comprehensive medical evaluation [4]. Recognizing the importance of CRAOs is essential for medical professionals and those under their care, as it facilitates swift identification, suitable interventions, and thorough long-term management of the condition.

Branch retinal artery occlusion (BRAO) is a vascular disorder characterized by the blockage of one or more branches of the central retinal artery, leading to acute, localized visual field loss [5]. BRAO occurs in about 40% of retinal artery obstructive diseases, and the visual prognosis is relatively better compared to CRAO [6]. Among patients with either permanent or transient BRAO, a majority of them initially experience a visual acuity of 20/40 or better (74% of permanent BRAO, 94% of transient BRAO and 73% of nonarteritic cilioretinal artery occlusion). After being treated, the visual acuity improved significantly [7]. While the visual prognosis for BRAO is generally favorable compared to CRAO, recurrent cases can result in significant retinal damage and vision loss. BRAO is clinically significant not only for its impact on vision but also because it serves as a potential marker for systemic vascular conditions, including cardiovascular and cerebrovascular diseases. Approximately 30% of individuals with acute CRAO and 25% of those with acute BRAO were found to have acute cerebral ischemia on MRI [8]. These significant rates highlight the importance of diagnosis and treating these conditions promptly to address both ocular and system risk factors effectively.

Atrial fibrillation (AFib) is one of the most common arrhythmias that can occur due to hypertension, heart diseases, heart valve problems, recent surgeries and other conditons [9–11] and may lead to suden cardiac death [12]. The incidence of AFib increases with age. During AFib, there are a multitude of pathophysiological changes that lead to progressive cardiac remodeling. One significant change is the increased intra-atrial pressure and progressive fibrosis of the atria, which leads to electric, contractile and structural remodeling [13,14]. These changes coupled with modification to the ion channel functionality and cellular changes contributes to the initiation and maintenance of atrial fibrillation [15]. There are several well studied mechanisms that play a significant role in the development and progression of atrial fibrillation: fibrosis, inflammation, oxidative stress, sedentary lifestyle, genetic factors, and dietary habits [16]. However, an important factor that is being further studied recently is the association between cryptogenic strokes and the increased risk of AFib detection. Studies underscore the importance of AFib detection and management in stroke patients [17,18].

The purpose of this article is to critically review the intricate relationship of atrial fibrillation in patients with retinal artery occlusion (RAO). Recent studies have underscored AFib as an emerging contributor to RAO risk, warranting further examination of their association. By synthesizing current research, this review aims to look into the prevalence of AFib in RAO patients and investigate potential associations between the two. Looking further into patient outcomes, this manuscript seeks to evaluate the clinical recommendation to extend further cardiac monitoring in RAO patients for AFib detection to mitigate cardiovascular toxicity and damage. Understanding this relationship is crucial for improving risk stratification, guiding treatment decisions, and developing more effective management strategies for RAO patients, ultimately contributing to better visual outcomes and reduced risk of subsequent adverse events.

## 2. Epidemiology of Retinal Artery Occlusion

Central retinal artery occlusion is a rare thromboembolic occlusion of the main artery that supplies blood to the eye. Studies conducted in several countries have provided detailed insights into the incidence of central retinal artery cclusion (CRAO). In Germany, the age-adjusted standardized incidence was found to be 2.7 per 100,000 person-years across all ages, escalating to 93.0 per 100,000 person-years for individuals over 80. Japanese research reported slightly lower rates, with 2.53 per 100,000 person-years overall and 47.5 per 100,000 person-years for those above 80. Korean data showed 2.00 per 100,000 person-years generally, increasing to 26.01 per 100,000 person-years in the over-80 age group. In the United States, the overall incidence was reported as 1.87 per 100,000 person-years. These figures consistently demonstrate a significant increase in CRAO incidence with age. Gender disparities were also noted, with Korean and Japanese studies revealing that men had 1.51 and 1.4 times higher incidence rates than women, respectively. CRAO not only severely impacts quality of life of the patients but also elevates their risk of subsequent cardiovascular and cerebrovascular events [19–21].

Branch retinal artery occlusion (BRAO) represents about 40% of all retinal artery obstructive diseases [6] and is caused by a blockage in one of the smaller branches of the central retinal artery, leading to localized ischemia and visual field defects. Research indicates that the incidence of BRAO ranges between 4.99 and 5 per 100,000 person-years globally, with an increasing prevalence observed in older populations [22].

Atrial fibrillation (AFib), conversely, is more common in the elderly population and continues to increase in prevalence. A 2024 study estimated that the current national prevalence of diagnosed AFib in the US is at least 10.55 million adults (95% CI: 10.48–10.62 million), comprising 4.48% (95% CI: 4.47%−4.49%) of the adult population. The proportion of patients with diagnosed AFib increased from 4.49% in 2005–2009 to 6.82% in 2015–2019 [23]. AFib prevalence estimates vary due to a less certain undiagnosed prevalence. A 2023 study estimated the total US prevalence of AFib (diagnosed and undiagnosed) in Q3 2015 was 5,628,000 cases where an estimated 591,000 cases (11%) were undiagnosed [24]. In a study with approximately 500,000 community-based patients, AFib incidence rates have increased significantly from 4.74 (95% CI, 4.58–4.90) to 6.82 (95% CI, 6.65–7.00) cases per 1,000 person-years between 2006 and 2018 (P <0.001). The fraction of AFib incidence in individuals who are 85 years or older increased from 12.6% in 2006 to 18.6% in 2017. In this community-based study, the rate of incidence for AFib has steadily increased from 2006 to 2017 [25].

## 3. Risk Factors for Retinal Artery Occlusion

Central retinal artery occlusion (CRAO) is associated with several cardiovascular and systemic risk factors [26]. Hypertension is a significant risk factor for retinal vascular occlusions, including CRAO. A study utilizing a multivariable-adjusted Cox model revealed that individuals with stage 2 hypertension had a significantly higher risk of retinal vascular occlusions compared to those with normal blood pressure. The hazard ratios (HRs) increased progressively with worsening hypertension stages, with stage 2 hypertension showing the highest risk (HR 1.32; 95% CI 1.20–1.46). While females appeared to have a higher risk across all blood pressure groups, this difference was not statistically significant. The study observed a trend of increased central retinal artery occlusion (CRAO) risk across all hypertensive categories compared to individuals with normal blood pressure. However, these findings lacked statistical significance, likely due to the limited number of CRAO events recorded: (HR, 0.94; 95% CI, 0.41–2.13) for the elevated BP group, HR, 0.99 (95% CI, 0.57–1.72) for the stage 1 hypertension group, and HR, 1.55 (95% CI, 0.91–2.62) for the stage 2 hypertension group [27,28]. Other factors such as tobacco smoking, hyperlipidemia, cardiac valvular disease, and other cardiovascular comorbidities were noted in CRAO cases compared with controls [29]. In addition to hypertension, type 2 diabetes mellitus (T2DM) has been investigated to have a causal relationship between T2DM and CRAO, as analyzed with various statistical methods. The inverse variance weighted (IVW) analysis revealed a significant association, suggesting that T2DM substantially increases CRAO risk (odds ratio [OR] =2.108, 95% confidence interval [CI]:1.221–3.638, P=7.423×10–3). This finding was corroborated by both maximum likelihood (OR=2.130, 95%CI:1.227–3.695, P=7.176×10–3) and weighted median approaches (OR=2.394, 95%CI:1.095–5.235, P=2.871×10–2), which yielded similar results. A validation dataset further supported these conclusions, reinforcing the robustness of the observed causal link. Importantly, the MR-PRESSO global test did not identify any outliers among the instrumental variables, lending additional credibility to the findings. Collectively, these results provide strong evidence for a causal role of T2DM in elevating CRAO risk [30,31].

Also, there is a clear trend between advancement of age and increasing retinal vascular occlusions, including CRAO. The majority of cases (70.3%) occurred in individuals aged 65–85 years, while only a small fraction (3.2%) affected those under 45 yeasr of age. Notably, 24.3% of cases were observed in the 45–65 years age group. The very elderly (85+ years) accounted for just 2.2% of cases, likely due to their lower representation in the IRIS Registry. Statistical analysis revealed strong positive correlations between age and onset of various retinal vascular occlusion subtypes, with Spearman coefficients ranging from 0.82 to 0.93. This correlation was markedly stronger than that observed for the overall set of IRIS Registry diagnoses (0.64), emphasizing the particular age-dependence of retinal vascular occlusions [32]. Other studies have found an increased risk of CRAO incidence rate of 10.08 per 100,000 from ages 80 to 84 compared to the 1.87 per 100,000 person-years found in the United States [33].

**Figure 1:**
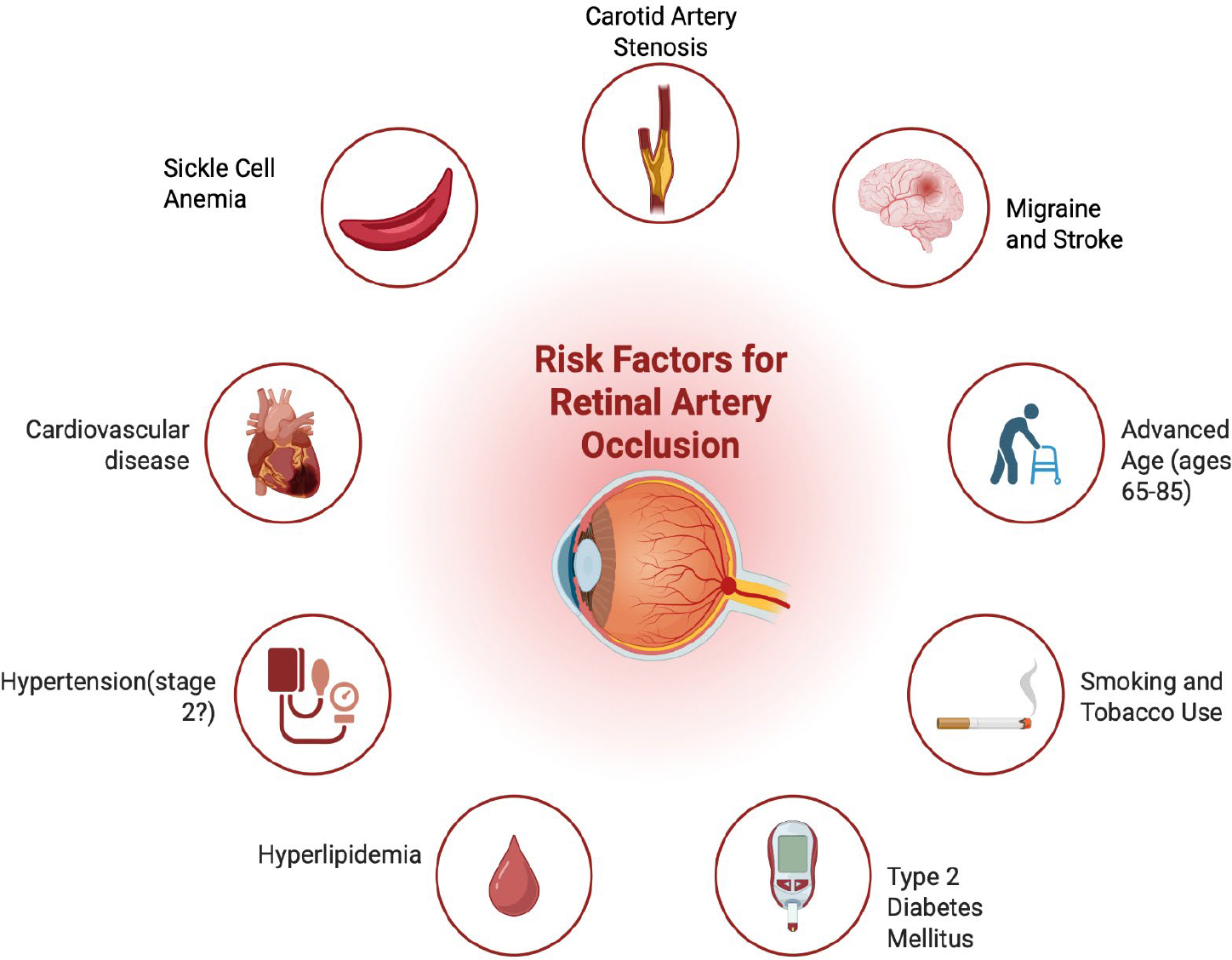
Risk factors for Retinal Artery Occlusions.

Branch retinal artery occlusions (BRAO) and central retinal artery occlusions (CRAO) share a number of risk factors including migraines, strokes, and carotid artery stenosis (Figure 1). Migraines with aura and without aura were studied as risk factors for retinal artery occlusions. The hazard ratio (HR) for developing any retinal artery occlusion (RAO) in individuals with migraines, compared to those without migraines, was 3.48 (95% CI 3.07–3.94; P <0.0001). This relationship was observed across all RAO subtypes, including central retinal artery occlusion (CRAO) with an HR of 1.62 (95% CI 1.15–2.28; P=0.004), branch retinal artery occlusion (BRAO) with an HR of 2.09 (95% CI 1.60–2.72; P <0.001), and other forms of RAO with an HR of 4.61 (95% CI 3.94–5.38; P <0.001). Furthermore, individuals experiencing migraine with aura had a greater risk of developing RAO compared to those with migraine without aura, with an HR of 1.58 (95% CI 1.40–1.79; P<0.001) [34]. When the risk of retinal vascular occlusions in association with migraines with and without aura was examined, the risk was found to be statistically significantly higher for both with aura (adjusted hazard ratio, 1.77; 95% CI, 1.58, 1.98) and without aura (aHR, 1.92; 95% CI: 1.64– 2.25) [35]. However, migraine was not found to be associated with CRAO when CRAO was listed as the primary diagnosis, with an adjusted hazard rate (aHR) of 1.15 (95% CI: 0.79–1.67). Although migraines and retinal artery occlusions were still associated, a significant association was observed when CRAO was considered in any diagnostic position with an aHR of 1.39 (95% CI: 1.08–1.78) [36].

In addition to migraines, retinal artery occlusions were linked to a history of strokes. The hazard ratio (HR) for experiencing a stroke following retinal artery occlusion (RAO), compared to after a hip fracture, was higher across all analyses. For all RAO cases, the HR was 2.97 (95% CI: 2.71–3.26; P< 0.001). Specifically, the HR for CRAO was 3.24 (95% CI: 2.83–3.70; P< 0.001), and for BRAO, it was 2.76 (95% CI: 2.43–3.13; P< 0.001) [37]. Retinal artery occlusions have also been associated with carotid artery stenosis. In a recent study of 45 patients in a case controlled study of patients with diagnosed retinal artery occlusion, 32 (88.9%) of the patients found plaques in the common carotid artery [38]. In a Cox regression analysis, ipsilateral carotid artery stenosis (ICAS) of over 70% occlusion was found to be significantly associated with a high risk of ischemic stroke after an acute retinal arterial ischemia (HR, 6.769; 95%CI:1.792–25.578, P=0.005) [39,40].

There are other factors including a high prevalence of thrombophilic risk factors [41–43], macular degeneration treatments - specifically brolucizumab [41] - and sickle cell anemia [44] that can also play a role in increasing the risk of developing retinal artery occlusions (Figure 1).

## 4. Risk Factors for Atrial Fibrillation

**Figure 2:**
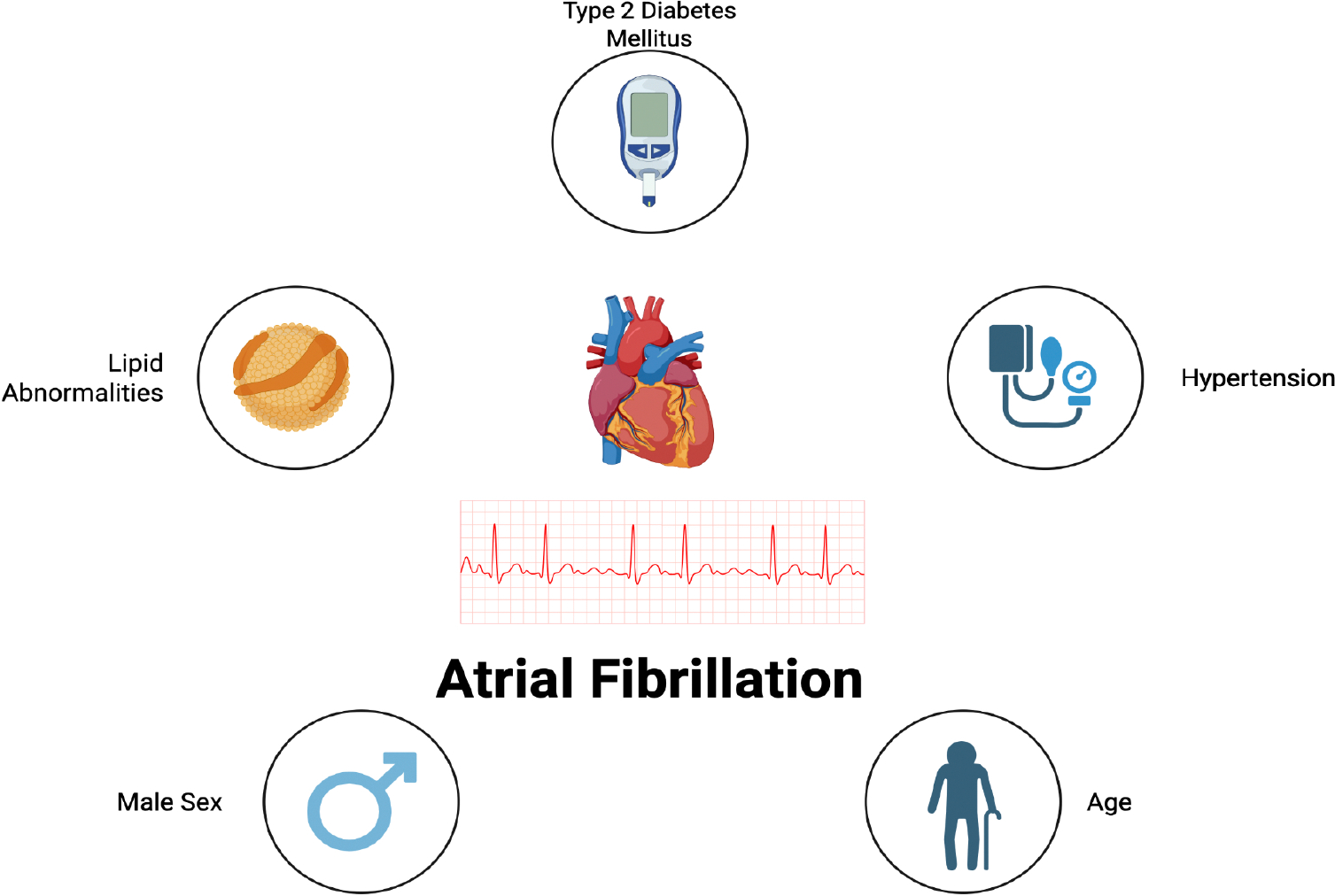
Risk factors for Atrial Fibrillation.

Similar to CRAO, atrial fibrillation (AFib) is associated with a multitude of cardiovascular risk factors (Figure 2). Hypertension is a common risk factor when it comes to a decline in cardiovascular health. Studies have found an increase in reported adjusted relative risk (RR) ratios in AFib patients who have hypertension. The summary RR with confidence intervals (CI) and the degree of heterogeneity (variation; I2) among the results of different studies for people with hypertension compared to a control was 1.50 (95% CI: 1.42–1.58, I2 = 98.1%, n = 56 studies) for developing AFib based on a meta-analysis conducted in 2023. Patients with a 20 mmHg increase in systolic blood pressure had a RR of 1.18 (95% CI: 1.16–1.21, I2 = 65.9%, n = 37 studies) and patients with a 10 mmHg increase in systolic blood pressure saw a RR of 1.07 (95% CI: 1.03–1.11, I2 = 91.5%, n = 22 studies). For higher blood pressures such as 180/110 mmHg, there was a 1.8–2.3 fold higher risk of AFib compared to those with a blood pressure of 90/60 mmHg [45,46]. These results suggest that elevated blood pressure and hypertension are key risk factors for AFib.

Along with hypertension, the role of cholesterol has been associated with the development of Afib (Figure 2). Some studies have described an inverse relationship between serum cholesterol levels and the risk of AFib. In a 2022 Swedish cohort study, high total cholesterol (TC) and low density lipoprotein cholesterol (LDL-c) were associated with a lower risk of AFib (HR = 0.61, 95% CI: 0.41 to 0.99, p = 0.013; HR = 0.64, 95% CI: 0.45 to 0.92, p = 0.016). Along with TC and LDL-C, higher Triglycerides (TG)/High Density Lipoprotein cholesterol (HDL-c) ratio, lower levels of high-density lipoprotein cholesterol (HDL-c) and apolipoprotein A-I (ApoA-I) were associated with an increased risk of new-onset AFib (HR ranging from 1.13; 95% CI: 1.07 to 1.19, p < 0.001 to 1.53; 95% CI: 1.12 to 2.00, p = 0.007) [47]. Additionally, low levels of remnant cholesterols (RC) were associated with an increased risk of AFib. When the quartile 1 and quartile 4 of RC levels from highest to lowest were analyzed, there was a hazard ratio of 1.396 (95% confidence interval [CI] 1.343–1.452) [48], supporting the conclusion that low RC levels were positively associated with an increased risk of AFib incidents independent of some cardiovascular risk factors.

Similarly with CRAOs, diabetes is also a prominent risk factor that has been associated with an increased risk of AFib. In a pooled prevalence of patients with a pre-existing diabetes mellitus, 26% of those patients had AFib. Diabetes mellitus was also associated with an increased risk of cardiovascular mortality in a multivariable-adjusted risk ratio (RR 1.46; 95% CI 1.34–1.58) [49]. Although the specific mechanism between diabetes mellitus and AFib still require further investigation, T2DM was linked with an estimated 40% greater risk of developing AFib compared to a control population (relative risk [RR] 1.39, 95% confidence interval [CI] 1.10 to 1.75, p for heterogeneity <0.001; RR of 1.34 95% confidence interval [CI] 1.07–1.68 after adjusting for publication bias) [50–52].

Along with diabetes, hypertension, cholesterol, and other modifiable factors, AFib has also been associated with age and sex based, non-modifiable factors (Figure 2). Specifically, among non-modifiable factors, increase in age and male sex are factors that are associated with an increase in risk of developing AFib [53,54]. Cardiometabolic factors consistently account for the largest proportion of incident AFib cases across all age groups, with hypertension being one of the leading modifiable factors. Understanding these risk factors is crucial for developing targeted prevention strategies and personalized management approaches for AFib. By addressing modifiable risk factors through lifestyle changes and appropriate medical interventions, it may be possible to reduce the incidence and progression of this common arrhythmia.

## 5. Association between Atrial Fibrillation and Retinal Artery Occlusion

AFib is a critical risk factor for embolic events that can lead to both retinal artery occlusions (RAO) and ischemic stroke, highlighting its systemic implications. In some cases, CRAO may precede acute ischemic stroke, particularly in younger patients, demonstrating the potential progression of cardioembolic events from retinal ischemia to cerebral infarction. Additionally, monocular blindness in patients with AFib should prompt urgent evaluation for possible internal carotid artery occlusion caused by embolism. Early diagnosis and timely interventions, such as mechanical thrombectomy, are essential to prevent further neurological damage and improve outcomes [55,56].

Studies have shown AFib to be significantly associated with RAOs [57]. Recent studies have shown an increase in prevalence of RAOs in patients with a history of AFib with the pooled prevalence of 11.5% (95% CI: 7.0–16.1, I2 = 96.3%). In addition, a meta-analysis found that AFib is significantly associated with retinal vessel occlusion (pooled OR = 2.24, 95% CI:2.07–2.43, I2 = 0.0%) [58,59]. Independent of any underlying ischemic heart diseases, AFib was also linked with an increased likelihood of developing retinal ischemic perivascular lesions (RIPLs). After adjusting for various confounding factors such as age, sex, smoking history, hypertension, diabetes, coronary artery disease, carotid stenosis, stroke and myocardial infarction, AFib was significantly associated with the presence of RIPLs (odds ratio = 1.91, 95% CI, 1.01–3.59). The association between RIPLs and AFib underlines the importance of doing a thorough cardiac work-up when a patient is diagnosed with a retinal vessel occlusion when detected on an optical coherence tomography scan of the macula [60].

Along with AFib being an independent risk factor for developing RAOs, some studies have reported an increase in rate of new AFib diagnosis after having a CRAO or RAO. Within two years following CRAO, the cumulative incidence of newly diagnosed AFib was 49.6% (95% CI: 37.4%–61.7%). Individuals with CRAO exhibited a significantly higher risk of developing AFib compared to controls (HR: 1.64; 95% CI: 1.17–2.31). In other studies, patients were put on Holter-ECG monitors to diagnose arrhythmias following a CRAO. Those studies found a high rate of newly diagnosed AFib, despite their minimal monitoring at only a single site. An interesting association was found while analyzing AFib following an episode of RAO. The rate of AFib in CRAO patients was similar to that observed in patients with ischemic stroke (HR: 1.01; 95% CI: 0.75–1.36) and the rate of new stroke compared to their matched control had a HR of 2.85 (CI 95%, 1.29–6.29) [61–63].

Along with AFib being associated with retinal strokes, patients with RAO have an increased risk of developing ischemic strokes. Patients with AFib and retinal arterial or venous occlusion are at an elevated risk for stroke, thromboembolism (TE), or transient ischemic attack (TIA). In a multivariate analysis, retinal artery occlusions were associated with a significantly higher risk of future stroke/TE/TIA (hazard ratio [HR] 1.39, 95% CI 1.08–1.79). AFib is a well known risk factor for the development of stroke/TE/TIA with a HR of 4.52 (95% confidence interval [CI] 4.44–4.60). In patients with RAO, the rate of stroke/TE/TIA jumps to a hazard ratio of 8.16 (95% CI 6.35–10.49) per 100 person-years [64–66].

These studies highlight the importance of cardiac work-up for high risk patients, and understanding the nuanced interconnectedness between these conditions that lead to better patient outcomes and early detection. Long term monitoring using implantable loop recorders for AFib could lead to pharmacotherapy to reduce the adverse effect of subsequent stroke and early intervention [67].

## 6. Diagnostic Procedures for Atrial Fibrillation

It is well established that the 12 lead electrocardiogram (ECG) is the gold standard in detecting and diagnosing AFib [68]. However, with the growing prevalence of smart watches, there has been a rise in wearable single lead ECGs. Some of those wearable 1 lead ECGs have been used to detect arrhythmias in patients. When the 12 lead ECG and the single lead ECG were compared, single-lead ECG demonstrated a sensitivity of 60.3% (95% CI: 47.7–72.0), a specificity of 97.2% (95% CI: 96.2–98.1), a positive predictive value (PPV) of 53.9% (95% CI: 42.1–65.5), and a negative predictive value (NPV) of 97.9% (95% CI: 96.9–98.6). In contrast, 12-lead ECG assessed by cardiac nurses showed significantly higher sensitivity at 97.1% (95% CI: 89.8–99.6), specificity at 100% (95% CI: 99.7–100), PPV at 100% (95% CI: 94.6–100), and NPV at 99.8% (95% CI: 99.4–100) [69,70]. The lack of specificity and sensitivity could be a result of the heterogeneity in the population and tools [71]. Additionally, there have been studies incorporating a deep densely connected neural network (DDDN) to detect AFib in 12 lead ECGs with high performance with an accuracy of 99.35 ± 0.26%, a sensitivity of 99.19 ± 0.31%, and a specificity of 99.44 ± 0.17% [72,73]. ECGs are an accurate non-invasive technique in AFib detection and diagnostics; however, some patients have episodes of AFib outside of the hospital. Insertable cardiac monitors (ICMs) are an invasive indication for long term monitoring for patients with unexplained cardiac arrhythmias. ICMs have proven to have a high diagnostic yield with a low serious adverse device and procedure related events [74–76].

## 7. Diagnostic Procedures for Retinal Artery Occlusions

Dilated fundoscopic examination is the gold standard for detecting retinal artery occlusions (RAO) as an initial diagnostic tool. The “cherry-red spot” found on the fovea coupled with a pale retina are characteristics of a central retinal artery occlusion (CRAO). Additionally, diagnostic tools such as fluorescein angiography and optical coherence tomography (OCT) are often used to confirm the diagnosis and assess the extent of retinal damage. OCT was found to be a highly effective, non-invasive tool for detecting abnormalities in retinal blood flow that was associated with RAO. OCT can provide vascular imaging used to identify vessel density changes in different retinal layers, making it a key diagnostic tool for both CRAO and BRAO [77–79]. Additionally, a questionnaire-based CRAO detection score was developed and validated, achieving an area under the curve (AUC) of 0.9 for diagnosing CRAO without fundoscopy. This method offers a rapid and non-invasive diagnostic tool, particularly useful in emergency settings [80].

## 8. Overview of Clotting (Coagulation cascade)

Hemostasis is a complex process involving multiple mechanisms that cause bleeding from blood vessels to stop through platelet plug formation at the injury site and activation of coagulation cascade. The cessation of bleeding can be grouped in 4 broad categories: vasoconstriction, formation of a “platelet plug”, coagulation cascade activation, and. “fibrin plug” (final clot). This series of enzymatic reactions allows platelets and fibrin to aggregate and regulate bleeding as the tissue heals [81].

Vasoconstriction occurs within the first 30 minutes of damage to the blood vessel where the extracellular matrix (ECM) and collagen become exposed to the blood elements. The ECM allows for the adhesion of those platelets through various factors to start the formation of a “platelet plug”. One notable factor is von Willebrand Factors (vWF) which binds to different glycoprotein receptors within the platelets causing them to adhere. After adhering, platelets undergo a change that causes them to release cytoplasmic granules such as ADP, serotonin, thromboxane A2, and other factors that allow for the activation and aggregation of those platelets that form the primary “platelet plug.” There are two major pathways that activate: Extrinsic and Intrinsic pathways. These pathways recruit multiple coagulation factors to lead to the activation of thrombin from prothrombin. Thrombin converts fibrinogen to fibrin, which is a crucial step for blood clot formation and is essential for hemostasis. Clots are eventually broken down by plasmin, which promotes blood flow in the damaged area after repair [81,82].

## 9. Potential Mechanisms of Thromboembolic Events

Hematocrit is the percentage of Red Blood Cells (RBCs) in the total blood volume. Increasing levels of hematocrit has been associated with an increase in blood viscosity, increased risk of thrombosis, and increased duration of interaction between a thrombus and platelets [83]. A large population-based study found that abnormal levels of hemoglobin, which is directly associated with hematocrit levels, was found in patients with a higher risk of AFib. The U-shaped relationship between hemoglobin levels and AFib risk emphasized the importance of maintaining hemoglobin levels to minimize AFib risk. Higher levels of hematocrit can increase blood viscosity, which is a risk factor for a thromboembolic event [84]. A recent study of a conditional logistic regression analysis revealed that hematocrit (Hct) levels of 40% or higher were linked to an increased risk of developing retinal artery occlusion (RAO). A forest plot demonstrated a potential non-linear dose-response relationship between Hct levels and ischemic vascular events in male patients. In individuals over 65 years old with six or more comorbidities, elevated Hct levels (≥40%) were associated with a higher likelihood of RAO. It is recommended that older patients with multiple comorbid conditions and elevated Hct levels be made aware of their potential risk for RAO, suggesting one possible mechanism of atrial fibrillation to increase risk of RAOs [85].

In addition to elevated hematocrit levels, One possible mechanism for the formation of multiple emboli may stem from turbulent blood flow through a narrowed vessel or diseased heart valve. This turbulence can dislodge unstable plaque fragments from the vessel wall, potentially resembling a comet where a primary nucleus is accompanied by smaller fragments shed simultaneously. However, this mechanism primarily applies to cases with carotid artery stenosis and is unlikely in cases where the carotid artery is completely occluded. In addition to direct embolism originating from carotid or cardiac lesions, indirect embolism via collateral circulation during significant hemodynamic changes associated with severe carotid artery obstruction (CAOD) may also play a role. Two collateral pathways are involved: intracranial and external collaterals. During hemodynamic shifts caused by severe carotid stenosis, compensatory blood flow from the contralateral internal carotid artery (ICA) through the circle of Willis can reverse direction, potentially mobilizing debris or cell fragments that form microemboli. These emboli are most likely generated at the distal segment of the ipsilateral ICA, where perfusion diminishes following proximal ICA occlusion. As compensatory intracranial flows restore perfusion, these emboli may travel downward to supply ophthalmic territories. This mechanism explains why patients with preserved collateral circulation and antegrade ophthalmic flow—observed via orbital color Doppler imaging—may experience retinal infarctions and visual symptoms without neurological deficits. The microemboli appear benign within cerebral territories but can cause visible damage in the retinal vasculature [86,87].

**Figure 3:**
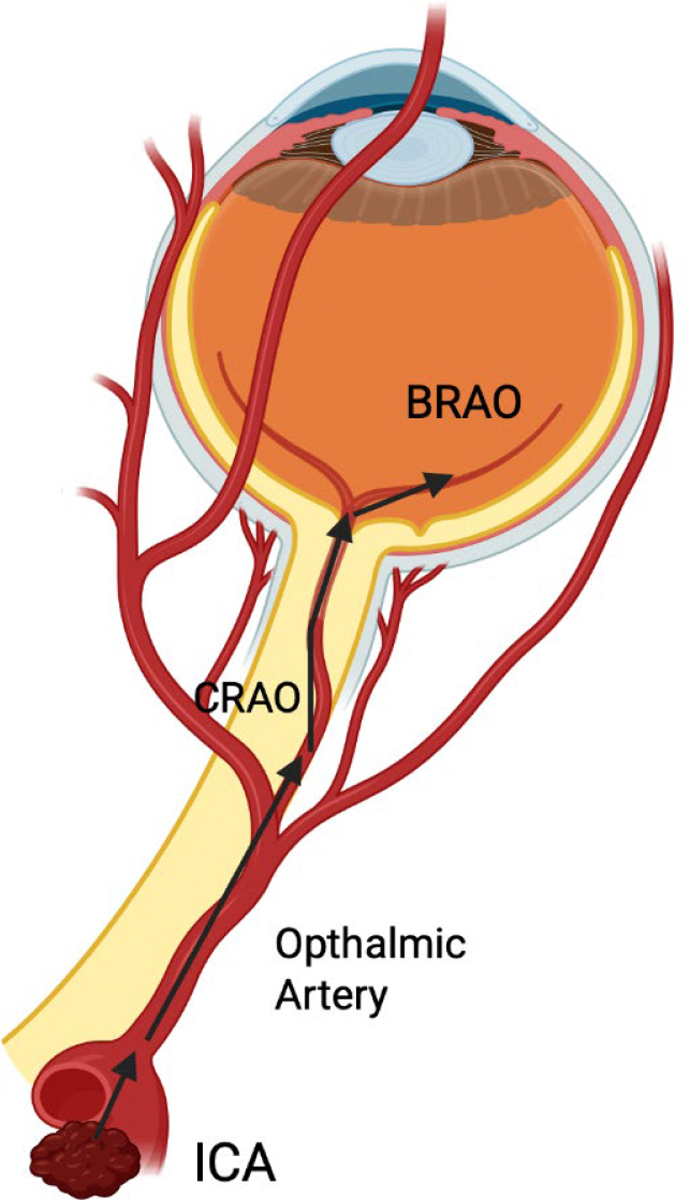
Potential pathway for fragmented plaque forming a clot. The clot travels from the Internal carotid artery to the ophthalmic artery and finally to the central retinal artery. If the emboli is small enough, it could spread into the branched retinal artery. BRAO, branch retinal artery occlusion; CRAO, central retinal artery occlusion; ICA, internal carotid artery.

Another potential mechanism could be the plaque buildup from internal carotid artery Stenosis. Emboli originating from the internal carotid artery are more likely to travel through the ophthalmic arteries rather than the middle cerebral arteries, while those coming from the left atrium tend to be directed into the middle cerebral artery instead of the ophthalmic artery (Figure 3). Emboli that develop due to carotid stenosis are often smaller than the thrombi that form in the atria. This difference may be because the high blood flow velocities across a significantly narrowed carotid artery can dislodge platelet-fibrin clots from the plaque surface early in their development, while they are still small. In contrast, thrombi in the atria, where blood flow is slower, can grow larger before eventually being released into the aortic arch. When a large clot moves up the internal carotid artery, it typically remains in the central flow and is mechanically unlikely to navigate the sharp turn into the ophthalmic artery (Figure 3). Smaller platelet-fibrin clots from carotid plaques, which originate near a stenosis, may be more prone to travel along the periphery of the bloodstream (boundary layer separation) and thus have a higher chance of entering the ophthalmic artery [88].

Studies between RAO and stroke have suggested a difference in pathophysiology between the two diseases. AFib has been linked as a stronger risk factor for brain events such as strokes compared to eye events such as RAO. AFib occurred more frequently in cerebral events compared to ocular events, both in prolonged cases (ischemic stroke vs. RAO: OR 3.6; 95% CI, 1.1–12) and in transient cases (cerebral TIA vs. amaurosis fugax: OR 2.9; 95% CI, 0.7–13). Additionally, AFib was more prevalent in prolonged than in transient episodes, whether in the brain (stroke vs. cerebral TIA: OR 3.3; 95% CI, 2.1–5.1) or in the eye (RAO vs. amaurosis fugax: OR 2.7; 95% CI, 0.4–16). However, in contrast, carotid artery disease is more strongly associated with eye events compared to brain events. Severe carotid artery disease on the same side was observed less often in cerebral events than in ocular events, both for prolonged cases (ischemic stroke compared to RAO: OR 0.6; 95% CI, 0.3–1.0) and for transient cases (cerebral TIA compared to amaurosis fugax: OR 0.4; 95% CI, 0.2–0.6) [88,89].

## 10. Treatment Strategy for Retinal Artery Occlusions

When it comes to treatment strategies for RAOs, there is no gold standard. Some clinicians take an aggressive approach through thrombolysis, while others incorporate a hyperbaric chamber to improve visual acuity [21,90]. For thrombolysis, there are various routes of administrations. A meta-analysis further compared the intravenous tissue plasminogen activator (i.v. tPA), intra-arterial urokinase (IAUK), and intra-arterial tPA (IAtPA) to see which strategy had better visual improvement. The meta-analysis found that the differences in visual improvement between the three were not statistically significant, but were statistically significant compared to the control. The findings indicated that intravenous tissue plasminogen activator (IVtPA) (OR: 5.78; 95% CI: 2.07–16.11), intra-arterial urokinase (IAUK) (OR: 2.78; 95% CI: 1.10–7.02), and intra-arterial tPA (IAtPA) (OR: 2.45; 95% CI: 1.04–5.77) were associated with greater visual improvement compared to control group. However, studies found that IV thrombolysis in RAO seems to be safe and effective within the first 4.5 hours of symptom onset. Compared to the control group, administering IVtPA within 4.5 hours of CRAO onset (OR: 8.87; 95% CI: 3.35 – 23.48) resulted in greater visual improvement than administration after 4.5 hours (OR: 3.09; 95% CI: 0.81–11.70). Additionally, while all evaluated thrombolytic strategies were linked to a higher risk of adverse events compared to the control, no significant differences were observed among these strategies. According to the surface under the cumulative ranking (SUCRA) analysis, IVtPA had the highest probability of achieving visual improvement (91.9%) but a comparatively lower probability of adverse events (60.1%) [91–93]. Along with IV and IA thrombolysis, a rare case of mechanical thrombectomy was performed for an isolated internal carotid artery (ICA) occlusion that was causing the patient monocular blindness. A balloon catheter was threaded through the cervical ICA to recanalize the occluded vessel. The procedure was completed with no complications and the patient reported significant visual acuity improvement immediately after the procedure [55].

In addition to thrombolysis, some patients undergo oxygen therapy through a hyperbaric chamber. Patients who underwent oxygen therapy demonstrated a significantly higher likelihood of visual improvement, approximately 5.61 times greater compared to the control group that did not receive oxygen therapy (OR = 5.61; 95% CI: 3.60–8.73; p < 0.01). No significant differences were found regarding the method of oxygen inhalation (χ^2^ = 0.18, df = 1, p = 0.67), combined therapy (χ^2^ = 0.21, df = 1, p = 0.64), or the type of RAO (χ^2^ = 0.06, df = 1, p = 0.81). However, both 100% oxygen (χ^2^ = 4.55, df = 1, p < 0.05) and hyperbaric oxygen (χ^2^ = 4.55, df = 1, p < 0.05) significantly enhanced visual acuity in RAO patients, with the most notable benefits observed within the first three months (χ^2^ = 5.76, df = 1, p < 0.05). The most effective treatment duration was over nine hours (χ^2^ = 6.58, df = 1, p < 0.05) [94,95].

## 11. Conclusions

Central retinal artery occlusion (CRAO) and branch retinal artery occlusion (BRAO) represent acute ophthalmic emergencies with profound implications for both ocular and systemic health. This comprehensive review underscores the multifactorial etiology of retinal artery occlusions, highlighting the significant roles of traditional vascular risk factors such as hypertension, diabetes mellitus, advanced age, and carotid artery disease. Notably, atrial fibrillation (AFib) has emerged as a critical and often under-recognized contributor to the risk of RAOs, paralleling its established role in cerebral ischemic events.

Epidemiological data consistently demonstrate an age-dependent increase in the incidence of both CRAO and AFib, with older adults facing a markedly higher risk for these conditions. The prevalence of AFib continues to rise, and its association with retinal vascular occlusions is increasingly evident. Studies reviewed in this manuscript reveal that AFib is not only a risk factor for the development of RAOs but is also frequently diagnosed following an episode of CRAO, suggesting a bidirectional relationship. This reciprocal association emphasizes the need for extended cardiac monitoring in patients presenting with retinal artery occlusion, as timely identification of paroxysmal or undiagnosed AFib can inform secondary prevention strategies and reduce the risk of future thromboembolic events, including stroke.

The pathophysiological mechanisms linking AFib and RAOs are complex, involving embolic phenomena that may originate from the heart or carotid arteries. Elevated hematocrit, increased blood viscosity, and turbulent blood flow further contribute to thrombus formation and embolization. The review also highlights that while carotid artery disease is more strongly associated with ocular ischemic events, AFib remains a potent risk factor for both retinal and cerebral vascular occlusions.

Diagnostic advances, including the use of 12-lead electrocardiograms, wearable cardiac monitors, and optical coherence tomography, have improved the detection of both AFib and retinal ischemic changes. However, there remains no universally accepted treatment for RAOs. Early interventions such as intravenous or intra-arterial thrombolysis and hyperbaric oxygen therapy may offer visual improvement if administered promptly, but their efficacy is highly time-dependent and associated with increased risk of adverse events.

Ultimately, the findings presented in this review advocate for a multidisciplinary approach to the management of RAO patients. Comprehensive cardiovascular evaluation, including prolonged cardiac monitoring for AFib, is essential for optimizing risk stratification and guiding therapeutic decisions. Increased awareness among clinicians and the public regarding the urgency of RAO symptoms and the potential systemic implications is critical for improving outcomes. By addressing modifiable risk factors and implementing timely diagnostic and therapeutic interventions, it is possible to reduce the burden of vision loss and prevent subsequent systemic vascular events in this high-risk population. The intricate interplay between AFib and retinal artery occlusions underscores the importance of integrated care pathways that bridge ophthalmology, cardiology, and primary care, ultimately striving for better visual and systemic health outcomes for affected individuals.

Ventricular function was assessed by echocardiography. Figure 2 shows that the MI-induced decrease in shortening fraction was sligthly but not significantly improved by Losartan while contractility was significantly preserved in GSK-LSD1-treated mice subjected to permanent coronary ligation (Figure 2A). The same beneficial and significant effect of GSK-LSD1 on ventricular function was observed in hearts with transient coronary ligation (Figure 2B).

## 12. Key Points

- CRAO and BRAO are caused by sudden interruptions in retinal blood flow, leading to varying degrees of vision loss and serving as markers for underlying systemic vascular diseases, with most patients presenting outside the optimal treatment window, highlighting the need for urgent recognition and comprehensive evaluation
- The incidence of CRAO and BRAO increases significantly with age, with CRAO rates rising sharply in individuals over the age of 80, and AFib prevalence also escalating in older populations, underscoring the age-dependent risk for both conditions
- Major risk factors for CRAO and BRAO include hypertension, type 2 diabetes mellitus, advanced age, hyperlipidemia, smoking, migraines, stroke history, and carotid artery stenosis, with these factors often overlapping and compounding the risk for retinal vascular events
- AFib shares many risk factors with RAO, such as hypertension, diabetes, lipid abnormalities, advanced age, and male sex, with hypertension and diabetes being particularly significant modifiable contributors to AFib development
- AFib is a significant independent risk factor for RAO, with studies showing increased prevalence of RAO in AFib patients and vice versa, and RAO patients are at higher risk for subsequent stroke and thromboembolic events, emphasizing the importance of cardiac evaluation and monitoring
- The 12-lead ECG remains the gold standard for AFib detection, but wearable single-lead ECGs and insertable cardiac monitors are increasingly used for prolonged monitoring, which improves AFib detection rates in at-risk populations
- Dilated fundoscopic examination is the primary diagnostic tool for RAO, with characteristic findings such as a “cherry-red spot,” while optical coherence tomography (OCT) and fluorescein angiography provide additional confirmation and assessment of retinal damage
- The coagulation cascade involves vasoconstriction, platelet plug formation, activation of coagulation factors, and fibrin clot formation, with elevated hematocrit and blood viscosity increasing the risk of thromboembolic events like RAO and AFib
- Thromboembolic events in RAO may result from emboli originating in the heart (especially with AFib) or carotid arteries, with turbulent blood flow and elevated hematocrit contributing to clot formation and embolization to the retinal circulation
- Specific mechanism surrounding RAOs are still largely unknown and should be a focus for future research
- There is no established gold standard for RAO treatment; early thrombolysis (intravenous or intra-arterial) and hyperbaric oxygen therapy may improve visual outcomes if administered promptly, but all carry risks, and visual improvement is most likely within a narrow time window after symptom onset.
